# Using eDNA to experimentally test ungulate browsing preferences

**DOI:** 10.1186/s40064-015-1285-z

**Published:** 2015-09-10

**Authors:** Ruth V. Nichols, Joris P. G. M. Cromsigt, Göran Spong

**Affiliations:** Molecular Ecology Research Group, Department of Wildlife, Fish, and Environmental Studies, Swedish University of Agricultural Sciences, Skogsmarksgränd, 90183 Umeå, Sweden; Department of Ecology and Evolutionary Biology, University of California Santa Cruz (UCSC), Santa Cruz, CA 95064 USA; Department of Zoology, Centre for African Conservation Ecology, Nelson Mandela Metropolitan University, NMMU, PO Box 77000, Port Elizabeth, 6031 South Africa; Fisheries, Wildlife, and Conservation Biology, College of Natural Resources, North Carolina State University, Raleigh, NC 27695-7646 USA

**Keywords:** Herbivory, DNA, Deer, Sweden

## Abstract

Large herbivores may affect ecosystem processes and states, but such effects can be difficult to quantify, especially within multispecies assemblages. To better understand such processes and improve our predictive ability of systems undergoing change, herbivore diets can be studied using controlled feeding trials (or cafeteria tests). With some wildlife, such as large herbivores, it is impractical to empirically verify these findings, because it requires visually observing animals in forested environments, which can disturb them from their natural behaviors. Yet, in field-based cafeteria trials it is nearly impossible to differentiate selection between herbivore species that forage on similar plants and make very similar bite marks. However, during browsing ungulates leave saliva residue which includes some buccal cells and DNA that can be extracted for species identification. Here we used a newly developed eDNA-based method (biteDNA) to test the browsing preferences of four sympatric ungulate species in the wild. Overall, food preferences varied between species, but all species strongly preferred deciduous over coniferous species. Our method allows the study of plant-animal interactions in multispecies assemblages at very fine detail.

## Background

In some areas, particularly across Europe and North America, ungulates are currently expanding their population sizes and ranges. These increases can cause dramatic changes to the landscape (Cote et al. 2004). Such changes can be difficult to predict, especially in multi-species assemblages containing several ungulate species with different feeding preferences. Such plant-herbivore relationships are important to understand in the context of trophic interactions, rewilding and species invasions. In forest ecosystems, for example, it is important to understand which trees ungulates prefer and how such preferences may affect plant succession and forestry operations. Dietary studies of such relationships are often done using feeding trials (also called cafeteria tests) where different foods are presented to an animal which then makes a choice as to what it will eat. However, cafeteria tests on wild ungulates are usually only practical where a single species occurs in the area. Ungulate bites tend to look the same and direct observations of the animals’ choices may be difficult or time consuming. Thus, many cafeteria studies are done with ungulates in captivity (Bergvall et al. 2006; Renaud et al. 2003; Shipley and Spalinger 1995). In some field studies, sites are assumed to be visited only by the predominant species, despite other species also being present (Jia et al. 1995; Milligan and Koricheva 2013; van Beest et al. 2010). DNA technology (Taberlet et al. 2012) makes it easier to study cryptic animal behaviors, such as herbivory. Herbivory by browsing herbivores can be cryptic when they are foraging in heavily forested environments with low visibility. We recently developed a new tool to study ungulate browsing based on the fact that when ungulates chew on shrubs or tree branches, they tend to leave behind enough saliva and environmental DNA (eDNA) for species identification (Nichols et al. 2012, 2015; Nichols and Spong 2014). Here, we expanded upon classical cafeteria tests by incorporating our browsed twig environmental DNA (biteDNA) method to differentiate the bites made by four ungulate species on artificial stands composed of three tree species. Thus, this is the first experimental test of foraging preferences using the biteDNA technique.

Our study was performed in an area where moose (*Alces alces*), roe deer (*Capreolus capreolus*), fallow deer (*Cervus dama*), and red deer (*Cervus elaphus*) co-occur. Moose and roe deer are known to browse extensively whereas red deer and fallow deer are mixed feeders, but fallow deer are known to fall closer to the grazer end of the spectrum (Hofmann 1989). In general, these ungulate species tend to overlap in their use of resources (Latham 1999; Mysterud 2000). During our study period (winter), with an abundance of snow, the animals only available food was browse and supplementary fodder provided by the landowners. In our cafeteria test, we mimicked stands of Scots pine (*Pinus sylvestris*), European aspen (*Populus tremula*) and goat willow (*Salix caprea*) to test the foraging preferences of these forest ungulates. After exposing our stands to the wild ungulates for two weeks, we collected browsed twig samples and used the biteDNA method to identify which ungulate species had browsed each twig.

## Results and discussion

144 out of 463 (31 %) browsed twig samples amplified DNA during PCR. All deer species were found to browse at our experimental plots. In our previous biteDNA studies, our amplification success has reached 80 % (Nichols and Spong 2014). We suggest that the freeze–thaw cycles during the study period are a reason behind the low amplification success rate. The samples that failed in our current study were evenly distributed across tree species and height classes. The freeze–thaw cycles may have broken off the buds on the shoots of the willow and aspen making them appear to be bites when they actually were not. Therefore, we caution other users of the biteDNA method to be aware of this. However, this also reveals a strength of the method we used; had we not used biteDNA, we might have overestimated browsing intensity and visitation rates to our cafeteria stations.

**Fig. 1:**
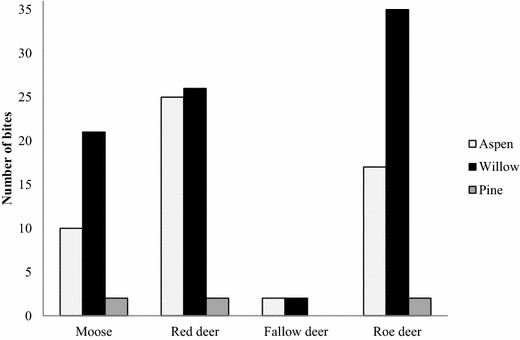
Distribution of bites on three tree species. The deer species were identified from saliva and DNA traces found on the browsed bites

Of the samples that identified deer species, most bites could be attributed to roe deer and red deer, followed by moose and fallow deer (Fig. 1). All ungulate species tended not to feed on pine (χ^2^ = 54.1, DF = 1, P < 0.0001). Six bites on pine were found whereas 84 and 54 bites on willow and aspen respectively were found (Fig. 1). Previous studies have indicated that moose prefer various species of willow and aspen over pine (Bergström and Hjeljord 1987; Franzmann and Schwartz 2007; Månsson et al. 2007) and that roe deer tend to prefer willow species over pine (Bergman et al. 2005). Aspen and willow species also tend to be highly palatable for red deer (Beschta and Ripple 2007) whereas pine is not as palatable (Elliott and Loudon 1987). Preferences between aspen and willow are less well studied in these species. In our study, moose and roe deer preferred willow over aspen (Moose χ^2^ = 3.9, DF = 1, P = 0.048; Roe deer χ^2^ = 7.1, DF = 1, P = 0.013). However, red deer showed no preference for willow or aspen (χ^2^ = 0.02, DF = 1, P = 0.89). We also found that, although fallow deer were abundant in this area, they did not browse our cafeteria trials very often. Four bites in total were sampled from fallow deer (Fig. 1). Thus, our results support and expand upon previous studies which suggest that these ungulate species share similar palatability rankings (i.e. willow ≥ aspen > pine). It is less clear how fallow deer rank these three species, but our results support fallow deer being a grazer since they did not choose to browse our sites very often.

## Conclusions

Cafeteria tests, like this, provide a way to empirically verify past studies and test ecological theories and this study is the first to experimentally test the browsing preferences of wild ungulates using biteDNA. This method is useful for studying cryptic browsing in particular. When presented with a choice to forage on willow and aspen versus pine (mimicking natural tree stands) all deer species tended to not use pine. In addition, we found that the deer species had different preferences for willow and aspen. Moose and roe deer preferred goat willow whereas red deer showed no clear preference. These preferences have implications in the area of forest management. The knowledge of deer preferences for certain trees may allow forest managers to better predict forest damages and successional stages based on the amount and species’ of trees available to ungulates.

## Methods

### Study area

We performed our cafeteria test near Nyköping in central Sweden (58°45′10″N, 17°00′28″E) in March 2010. This area holds relatively high densities of ungulates (Table 1). Landscape features included lakes, wetlands, forest plantations of differing ages and agricultural lands. The average temperature during March in this area was −0.5 °C and snow depth was 50-75 cm (SMHI 2010).

**Table 1:** Density estimates according to offtake levels and observations by professional hunters (numbers per 1000 hectares) (Svenska Jägareförbundet, pers comm)

Species	Density
Moose	7
Red deer	15
Fallow deer	140
Roe deer	30
Total	192

### Cafeteria test

**Fig. 2:**
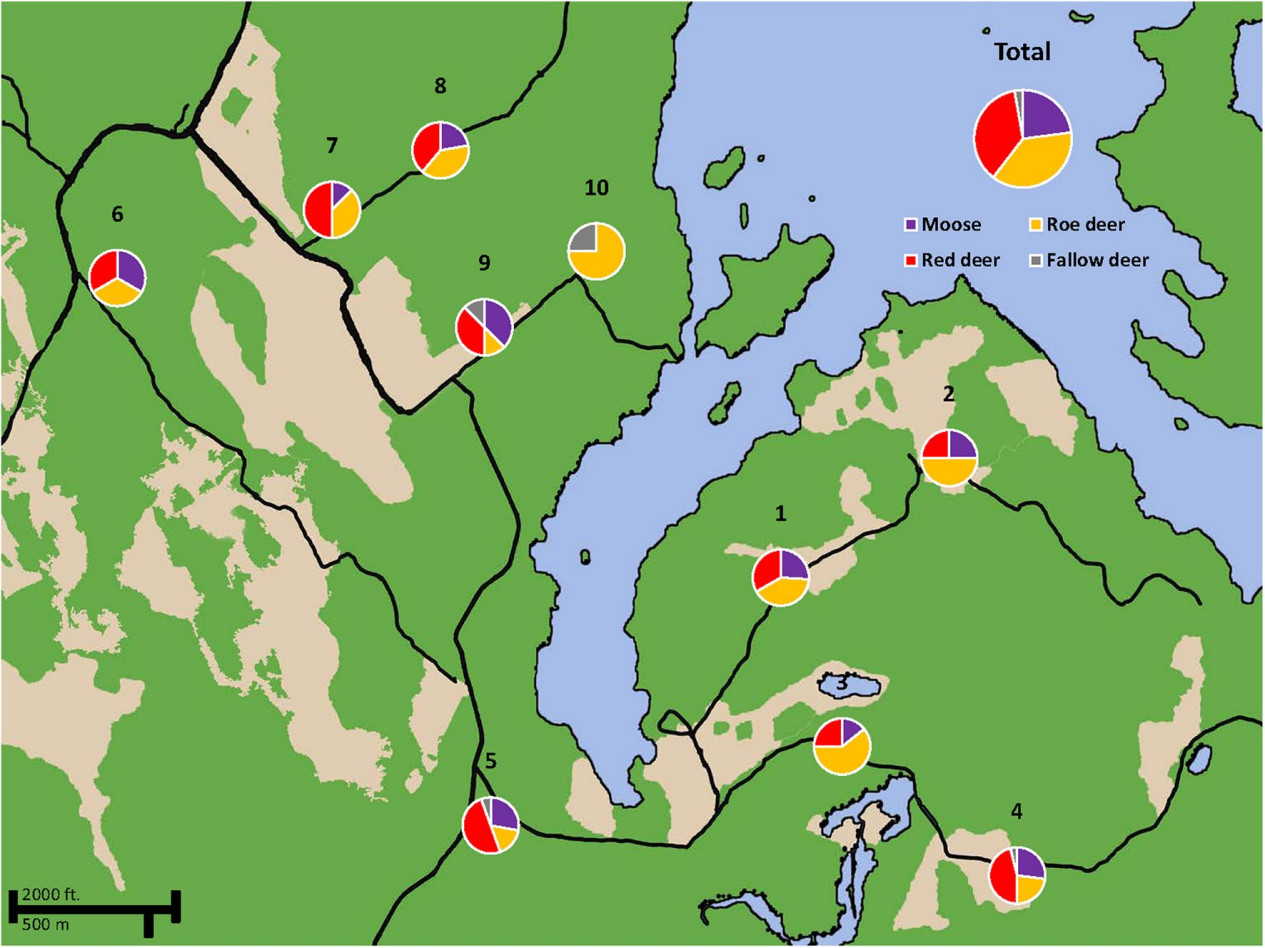
Map of the study area with pie charts showing proportions of bites found and identified to the responsible deer species. *Green areas* on the map indicate areas with relatively higher tree densities. The *lighter*, *tan areas* indicate areas either devoid of trees or with relatively lower densities of trees. The *blue area* is a lake

To test browsing preferences, we obtained 100 unbrowsed, approximately 2 m tall branches of goat willow and European aspen and trees of Scots pine, respectively. This test was done during winter when the willow and aspen branches had no leaves. Two field workers created 100 test stations consisting of bundles composed of one branch of goat willow, one branch of aspen and one pine tree. These bundles were tied together to posts and existing tree trunks out in the field (all were older trees offering little or no alternative forage). We had 10 sites consisting of 10 stations each. Sites were approximately 0.5–7 km apart. Stations were also approximately 5–10 m apart (Fig. 2). After 2 weeks we returned to collect browsed bites. We systematically collected bites from low, medium and high heights to reduce any bias in height and we collected between 0 and 4 bites from each tree species in proportion to browsing intensity. For example, if a tree was heavily browsed at all available heights, we collected 1 bite from low heights (0–75 cm), 1 bite from medium heights (75–125 cm), and 1 bite from high heights (125–200 cm). We collected a fourth bite from extremely browsed trees, and it was collected at a random height. We collected a total of 463 samples. The cost of the eDNA method prevented us from collecting all bites. A full cost sample including labor, equipment depreciation, plastics and chemicals is in the order of 40 USD.

### biteDNA protocol

Collection of all browsing samples followed the protocols in Nichols et al. (2012). Briefly, the top 1.5–2 cm of browsed twigs were clipped into two ml centrifuge tubes filled with phosphate-buffered saline solution. We transported these samples at ambient temperatures until they reached our lab (within 1 week) and were then stored at −20 °C until DNA extraction. DNA extraction followed the final (robotic) extraction protocol and all PCR amplifications and fragment analyses followed the protocols found in Nichols et al. (2012).

### Statistics

To test the preferences for tree species we used χ^2^ tests to see if the distribution in numbers of bites was significantly different from an even distribution. By ‘even distribution’ we mean that if a deer made 50 bites total, an even distribution that indicated no preference between willow and aspen would have been 25 bites on willow and 25 bites on aspen. We used JMP 9.0 for these analyses (SAS Institute Inc., Cary, NC, USA).

## References

[CR1] Bergman M, Iason G, Hester A (2005). Feeding patterns by roe deer and rabbits on pine, willow and birch in relation to spatial arrangement. Oikos.

[CR2] Bergström R, Hjeljord O (1987). Moose and vegetation interactions in northwestern Europe and Poland. Swed Wildl Res Suppl.

[CR3] Bergvall UA, Rautio P, Kesti K, Tuomi J, Leimar O (2006). Associational effects of plant defences in relation to within- and between-patch food choice by a mammalian herbivore: neighbour contrast susceptibility and defence. Oecologia.

[CR4] Beschta RL, Ripple W (2007). Wolves, elk, and aspen in the winter range of Jasper National Park, Canada. Can J For Res.

[CR5] Cote SD, Rooney TP, Tremblay JP, Dussault C, Waller DM (2004). Ecological impacts of deer overabundance. Annu Rev Ecol Evol Syst.

[CR6] Elliott S, Loudon A (1987). Effects of monoterpene odors on food selection by red deer calves (Cervus elaphus). J Chem Ecol.

[CR7] Franzmann AW, Schwartz CC (2007) Ecology and management of the North American moose. Univ Pr of Colorado

[CR8] Hofmann RR (1989). Evolutionary steps of ecophysiological adaptation and diversification of ruminants: a comparative view of their digestive system. Oecologia.

[CR9] Jia J, Niemelä P, Danell K (1995). Moose Alces alces bite diameter selection in relation to twig quality on four phenotypes of Scots pine Pinus sylvestris. Wildl Biol.

[CR10] Latham J (1999). Interspecific interactions of ungulates in European forests: an overview. For Ecol Manage.

[CR11] Månsson J, Kalén C, Kjellander P, Andrén H, Smith H (2007). Quantitative estimates of tree species selectivity by moose (Alces alces) in a forest landscape. Scand J For Res.

[CR12] Milligan HT, Koricheva J (2013). Effects of tree species richness and composition on moose winter browsing damage and foraging selectivity: an experimental study. J Anim Ecol.

[CR13] Mysterud A (2000). Diet overlap among ruminants in Fennoscandia. Oecologia.

[CR14] Nichols RV, Spong G (2014). Ungulate browsing on conifers during summer as revealed by DNA. Scand J For Res.

[CR15] Nichols RV, Königsson H, Danell K, Spong G (2012). Browsed twig environmental DNA: diagnostic PCR to identify ungulate species. Mol Ecol Res.

[CR16] Nichols RV, Cromsigt JGM, Spong G (2015). DNA left on browsed twigs uncovers bite-scale resource use patterns in European ungulates. Oecologia.

[CR17] Renaud PC, Verheyden-Tixier H, Dumont B (2003). Damage to saplings by red deer (Cervus elaphus): effect of foliage height and structure. For Ecol Manage.

[CR18] Shipley LA, Spalinger DE (1995). Influence of size and density of browse patches on intake rates and foraging decisions of young moose and white-tailed deer. Oecologia.

[CR19] SMHI (2010) Swedish Meteorological and Hydrological Institute. http://www.smhi.se. Accessed 15 Jan 2013

[CR20] Taberlet P, Coissac E, Hajibabaei M, Rieseberg LH (2012). Environmental DNA. Mol Ecol.

[CR21] van Beest FM, Gundersen H, Mathisen KM, Milner JM, Skarpe C (2010). Long-term browsing impact around diversionary feeding stations for moose in Southern Norway. For Ecol Manage.

